# Plasma levels of M-CSF and VEGF in laboratory diagnostics and differentiation of selected histological types of cervical cancers

**DOI:** 10.1186/s12885-019-5558-8

**Published:** 2019-04-29

**Authors:** Iwona Sidorkiewicz, Monika Zbucka-Krętowska, Kamil Zaręba, Emilia Lubowicka, Monika Zajkowska, Maciej Szmitkowski, Ewa Gacuta, Sławomir Ławicki

**Affiliations:** 1 Department of Reproduction and Gynecological Endocrinology, Medical University of Bialystok, 15-276 Bialystok, Poland; 2 Department of Gynecology and Gynecologic Oncology, Medical University of Białystok, 15-276 Bialystok, Poland; 3 Department of Esthetic Medicine, Medical University of Bialystok, 15-089 Bialystok, Poland; 4 Department of Biochemical Diagnostics, Medical University of Bialystok, 15-269 Bialystok, Poland; 5 Department of Perinatology, Medical University of Bialystok, 15-276 Bialystok, Poland; 6 Department of Population Medicine and Civilization Diseases Prevention, Medical University of Bialystok, 15-269 Bialystok, Poland; 7 Present address: Clinical Research Centre, Medical University of Bialystok, 15-276 Bialystok, Poland

**Keywords:** M-CSF, VEGF, Cervical cancer, Serum marker, Tumor marker, Squamous cell carcinoma, Adenocarcinoma

## Abstract

**Background:**

The search of useful serum biomarkers for the early detection of cervical cancers has been of a high priority. The activation of Macrophage-Colony Stimulating Factor (M-CSF) and Vascular Endothelial Growth Factor (VEGF) is likely involved in the pathogenesis and spread of cancer. We compared the plasma levels of M-CSF and VEGF to the ones of commonly accepted tumor markers CA 125and SCC-Ag in three groups of patients: 1. the cervical cancer group (patients with either squamous cell carcinoma or adenocarcinoma); 2. the cervical dysplasia group; 3. the control group.

**Methods:**

This cohort study included 100 patients with cervical cancer and 55 patients with cervical dysplasia. The control group consisted of 50 healthy volunteers. The plasma levels of VEGF and M-CSF were determined using ELISA, while CA 125 and SCC-Ag concentrations were obtained by the chemiluminescent microparticle immunoassay (CMIA).

**Results:**

The median levels of M-CSF and VEGF as well as CA 125 and SCC-Ag in the entire group of cervical cancer patients, were significantly different compared to the healthy women group. In case of both the squamous cell carcinoma and the adenocarcinoma groups, plasma levels of M-CSF and VEGF were higher compared to the control group. No significant differences in the studied parameters between the squamous cell carcinoma and the adenocarcinoma group were observed. The highest sensitivity and specificity were obtained for VEGF (81.18 and 76.00%, respectively) and SCC-Ag (81.18%; 74.00%) in the squamous cell carcinoma group and for VEGF (86.67%; 76.00%) in the adenocarcinoma group. The area under the ROC curve for VEGF was the largest in the adenocarcinoma group followed by the squamous cell carcinoma group (0.9082 and 0.8566 respectively).

**Conclusions:**

Obtained results indicate a possible clinical applicability and a high diagnostic power for the combination of MSC-F, VEGF, CA 125 and SCC-Ag in the diagnosis of both studied types of cervical cancer.

## Background

Cervical cancer remains one of the most common type of cancer worldwide and the third cause of death among women in developing countries [1]. What is important, it is characterized by a long period of preclinical disease progression through a number of well-defined pre-cancerous cervical intraepithelial neoplasia (CIN) grades I through III [2]. It has been established that the global introduction of cervical cytology as the preferred screening method resulted in a significant decrease of cervical cancer incidence [3, 4]. Nevertheless, malignancies of the cervix remain an important health issue in the developing countries. Pap smears, although a gold standard in the prevention of cervical cancer, has insufficient sensitivity. The high rate of false negative results of cervical cytology leads to the misdiagnosis of many cervical cancer patients [5]. Currently, an additional HPV (Human Papilloma Virus) - genotyping screening program has been introduced to improve the diagnostic sensitivity [6–8]. However, due to high cost- inputs, new markers are still being sought for early diagnosis of cervical cancer [9–11]. Despite the aggressive operative and systemic treatment procedures, the outlook remains unfavorable for patients with advanced stages of the disease [12]. Therefore, there is an important clinical implication for early diagnosis of cervical cancer and evaluation of overall prognosis [13].

Vascular Endothelial Growth Factor (VEGF) family constitutes one of the most important signaling pathways associated with angiogenesis in the development of malignant disease. VEGF, a dimeric glycoprotein of 34–42 kDa, is expressed by a variety of normal cells and malignant tumors, where it can be secreted by the tumor cells themselves or by stromal cells [14]. In particular, its expression is demonstrated to be correlated to hypoxia [15]. Initial studies have found that anti- VEGF treatment induces vascular regression and consequently is effective in inhibiting tumor growth and metastasis [16, 17]. It has been confirmed by other studies that VEGF plays an important role in the development of breast [18–21], reproductive organ [22–24] and ovarian cancer [25–27].

As the major steps in the development of the studied cervical cancer histotypes are commonly known, the role of chronic inflammation process in cancer invasion has been extensively studied. However, there is still lack of plasma markers identifying the biological processes leading to advanced disease in an early clinical grading [28]. The Macrophage-Colony Stimulating Factor (M-CSF) is one of the cytokines called hematopoietic growth factors (HGFs) and regulates the macrophage homeostasis, primarily the growth, differentiation and function. It has been demonstrated that overexpression of various chemotactic and growth factors such as M-CSF leads to recruitment of tumor-associated macrophages (TAMs) in different types of cancers and can stimulate cancer cell proliferation and/or migration. Studies also indicate crucial role of M-CSF in tumor development, while M-CSF receptor (M-CSFR) signaling inhibitors have the potential to effectively suppress the primary tumor growth, tumor angiogenesis and disorganize extracellular matrix [29–31].

The aim of this study was to determine the plasma levels of M-CSF and VEGF in comparison to known tumor markers CA 125 (Cancer Antigen 125) and SCC-Ag (Squamous Cell Carcinoma Antigen) in patients with 2 different types of cervical cancer (squamous cell carcinoma and adenocarcinoma) in relation to the patients with cervical dysplasia and the control group consisting of healthy subjects.

## Methods

### Human subjects

The study comprised 85 patients with squamous cell carcinoma, 15 patients with adenocarcinoma and 55 patients with cervical dysplasia who were referred to the Department of Gynaecology, Bialystok Medical University Teaching Hospital, Poland (Table 1). The clinical staging was determined in accordance with 2014 International Federation of Gynecology and Obstetrics (FIGO) criteria in all cases [32]. Histological evaluation of the obtained samples was performed concordantly with the recent recommendations from the College of American Pathologists and the American Society for Colposcopy and Cervical Pathology [33]. Ethical approval for the study was obtained originally from the local Ethics Committee at the Medical University of Bialystok, Poland (R-I-002/239/2014). Before the study entry all patients participating in the study read and signed forms of informed consent specifically approved for this project by the Ethics Committee. The groups were homogeneous and did not differ regarding the menopausal status. No inflammation process was confirmed by laboratory tests (CRP, leukocytosis) and physical examination. All samples were taken prior to any treatment and any medication was not accepted at the time of blood sample collection. The control group included 50 healthy and untreated women (aged 22–61 years). The included patients were not referred from other medical centers. The gynecological exam, a reproductive organ ultrasound scan and a cervical smear were performed.

**Table 1:** Characteristics of cervical cancer, dysplasia patients and control group

Studygroup			Number of patients
Examined Groups	Cervical cancer patients	*Squamous cell carcinoma*	85
		*Adenocarcinoma*	15
	Median age (range)		46 (25-61)
	Tumor stage	I	32
		II	33
		III+IV	35
	Menopausal status:		
	- premenopausal		78
	- postmenopausal		22
	Cervical dysplasia patients		55
	Median age (range)		44 (23-60)
	CIN stage	CIN1	15
		CIN2	20
		CIN3	20
	Menopausal status:		
	- premenopausal		33
	- postmenopausal		22
Control Group	Healthy women		50
	Median age (range)		42 (22-61)
	Menopausal status:		
	- premenopausal		40
	- postmenopausal		10

### Plasma collection and storage

Venous blood was collected from each patient into a heparin sodium tube as previously [34, 35], centrifuged at 3500 rpm for 20 min to obtain plasma samples, and stored until assayed.

### Measurement of M-CSF, VEGF, CA 125 and SCC-ag

**Fig. 1:**
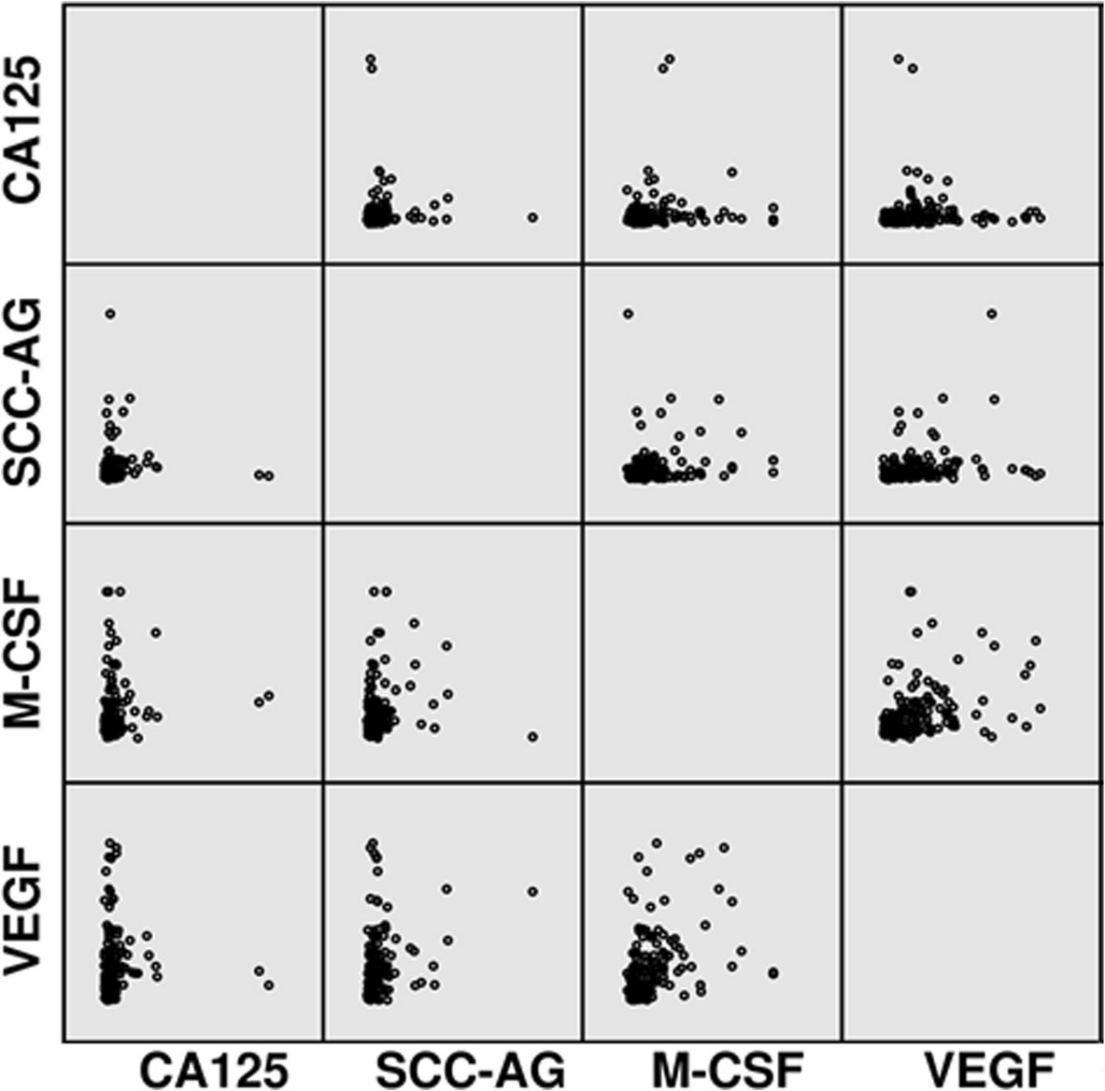
Scatterplots of the studied parameters

The tested cytokines (M-CSF, VEGF) were measured in plasma with enzyme-linked immunosorbent assay (ELISA) (Quantikine Human M-CSF Immunoassay; R&D systems, Abingdon, United Kingdom), according to the manufacturer’s protocols (Fig.1). Plasma concentrations of CA 125 and SCC-Ag were measured by chemiluminescent microparticle immunoassay (CMIA) (Abbott, Chicago, IL, USA). Duplicate samples were assessed for each standard, control, and sample. The value of intra- and inter- assay CVs were calculated by the manufacturers and enclosed in the reagent kits. The assay does not exhibit cross-reactivity or interference with numerous human cytokines and other growth factors [34].

### Statistical analysis

Statistical analysis was performed by IBM SPSS Statistics for Windows 20.0 (IBM Corp., Armonk, NY, USA). The Shapiro-Wilk test for preliminary assessment revealed that the cytokine and tumor marker levels did not follow normal distribution. Consequently, statistical analysis between the groups was performed by using the U-Mann Whitney test, the Kruskal-Wallis test and a multivariate analysis of various data by the post-hoc Dwass-Steele-Crichlow-Flinger test [36, 37]. The data were presented as a median and a range. An exploratory, hypothesis generating study was conducted where associations were deemed suggestive if the *p*- value was less than 0.05. Diagnostic sensitivity (SE) and specificity (SP) were calculated by using cut-off values which were calculated by the Youden’s index (as a criterion for selecting the optimum cut-off point) [38] and for each of the tested parameters were as follows: M-CSF – 397.65 pg/mL; VEGF – 88.46 pg/mL; CA 125–39.30 U/mL; SCC-Ag – 1.25 ng/mL.To estimate total diagnostic value of more than one variable, their linear combinations based on logistic regression model were calculated, and these one-dimensional combinations were used in ROC (Receiver Operator Characteristic) analyses (Table 2). For the diagnostic performance (sensitivity, specificity) and ROC curve, only healthy subjects were used as the control group. All the calculations related to ROC analyses including the construction of the ROC curves were performed using Microsoft Excel 2010 software following the methodology described in literature.

**Table 2:** The linear combinations of studied parameters

Variables	Linear combination
CA125, MCSF	-1.281 - 0.00677 * CA125 + 0.00531 * MCSF
CA125, VEGF	-1.719 - 0.00353 * CA125 + 0.0261 * VEGF
SCCAG, MCSF	-4.428 + 3.22 * SCCAG + 0.00475 * MCSF
SCCAG, VEGF	-4.438 + 2.80 * SCCAG + 0.0244 * VEGF
CA125, SCCAG, MCSF	-4.539 - 0.00758 * CA125 + 3.26 * SCCAG + 0.00547 * MCSF
CA125, SCCAG, VEGF	-4.390 - 0.00385 * CA125 + 2.81 * SCCAG + 0.0250 * VEGF

## Results

Table 3 presents the medians and ranges for the investigated plasma levels of M-CSF, VEGF, CA 125 and SCC-Ag in studied groups (Table 3). The medians of M-CSF, VEGF, CA 125 and SCC-Ag (500.55 pg/ml,142.00 pg/ml,17.60 U/ml, and 1.20 ng/ml respectively) in cervical cancer indicated suggestive differences when compared to the healthy women group (251.50 pg/ml;45.80 pg/ml;11.70 U/ml and 0.75 ng/ml respectively) (*p* < 0.05). In case of the squamous cell carcinoma and adenocarcinoma groups, differences in plasma levels of M-CSF and VEGF were observed. CA 125 and SCC-Ag median values were statistically different between the squamous cell carcinoma patients and the healthy patients. In contrast, there were no significant differences observed in CA 125 concentrations in the adenocarcinoma patients compared to the control group. Additionally, levels of all tested markers in the squamous cell carcinoma group and total cervical cancer group were notably higher than in the dysplasia group (medians for M-CSF, VEGF, CA 125 and SCC-Ag were 312.34 pg/ml, 62.60 pg/ml, 14.90 U/ml and 0.8 ng/ml respectively) (*p* < 0.001). No statistical differences were observed between the concentration of any of the tested parameters in patients with CIN I, CIN II, CIN III and the control group. Only M-CSF median was significantly higher in the cervical dysplasia group compared to the control group. We also did not note any difference in plasma level of tested parameters between the two studied groups of cervical cancer.

**Table 3:** Plasma levels of tested parameters and CA 125 and SCC-Ag in patients with cervical cancer, dysplasia patients and in control group (median and range)

		M-CSF (pg/mL)	VEGF (pg/mL)	CA 125 (U/mL)	SCC-Ag (ng/mL)
Cervical cancer	*Squamous cell carcinoma*	510.55^a/b/e^ 102.15–2513.75	140.20^a/b/c/e^ 11.80–577.22	17.99^a/e^ 4.40–120.10	1.20 ^a/b/c/e^ 0.50–14.10
	*Adenocarcinoma*	442.41^a/b^ 95.23–1696.65	158.88^a/b/e^ 56.76–615.50	15.50 6.34–77.41	1.30^a^ 0.70–7.10
	Total	500.55 ^a/e^ 95.23–2513.75	142.00 ^a/e^ 11.80–615.50	17.60 ^a/e^ 4.40–120.10	1.20 ^a/e^ 0.50–14.10
CIN grade	CIN I	132.40 11.80–615.50	47.40 28.20–407.22	132.40 11.80–615.50	0.74 0.58–0.87
	CIN II	136.90 28.92–395.60	48.94 16.90–467.10	136.90 28.92–395.60	0.70 0.55–1.00
	CIN III	199.00 44.50–598.50	111.50 27.12–426.86	199.00 44.50–598.50	0.85 0.65–1.40
	Total	312.34 ^a^ 126.20–1830.20	62.60 16.90–467.10	14.90 2.53–78.30	0.80 0.30–5.20
Control group		251.50 ^d/e^ 119.63–935.29	45.80 ^d^ 11.20–194.50	11.70 3.50–366.00	0.75 0.40–1.60

CA 125: cancer antigen 125; SCC-Ag: squamous cell carcinoma antigen; M-CSF: macrophage-colony stimulating factor; VEGF: vascular endothelial growth factor

^a^Statistically significant when compared with controls

^b^Statistically significant when compared with patients with CIN I

^c^Statistically significant when compared with patients with CIN II

^d^Statistically significant when compared with patients with CIN III

^e^Statistically significant when compared with patients with total CIN group

**Fig. 2:**
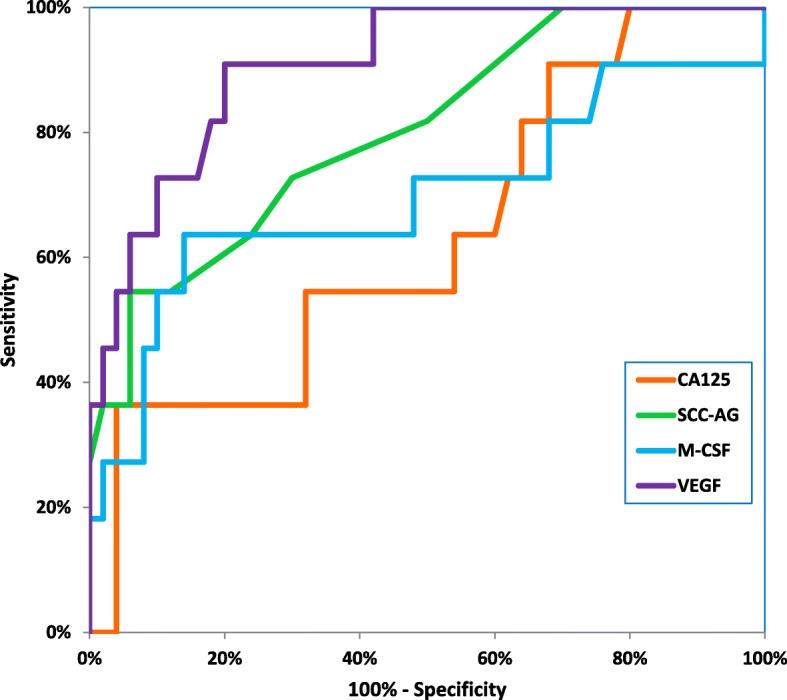
Diagnostic criteria of ROC curve for tested parameters in adenocarcinoma cervical cancer group

**Fig. 3:**
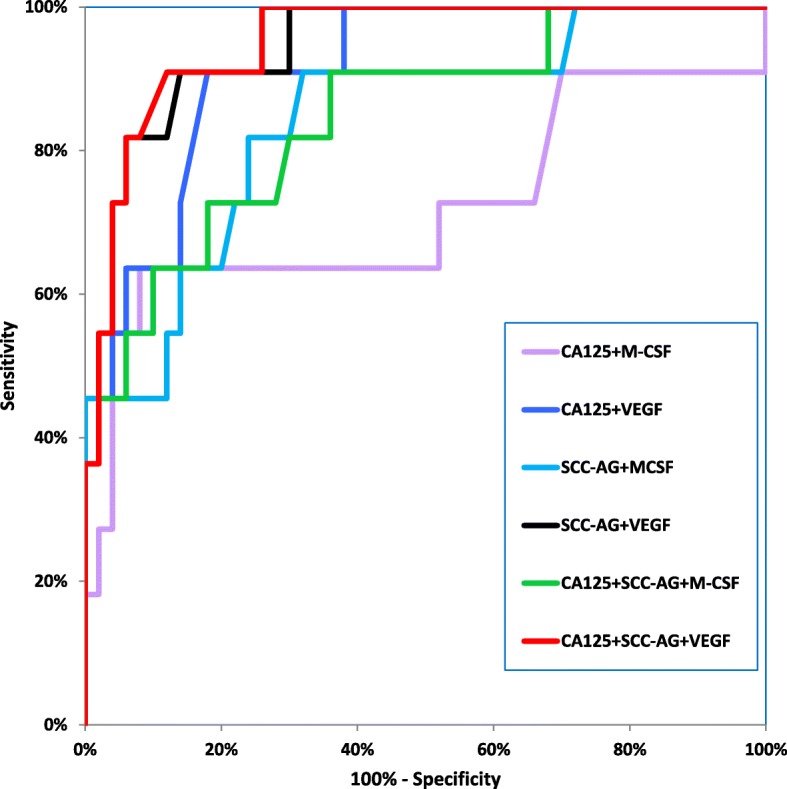
Diagnostic criteria of ROC curve for tested parameters in combination with CA 125 and SCC-Ag in adenocarcinoma cervical cancer group

**Fig. 4:**
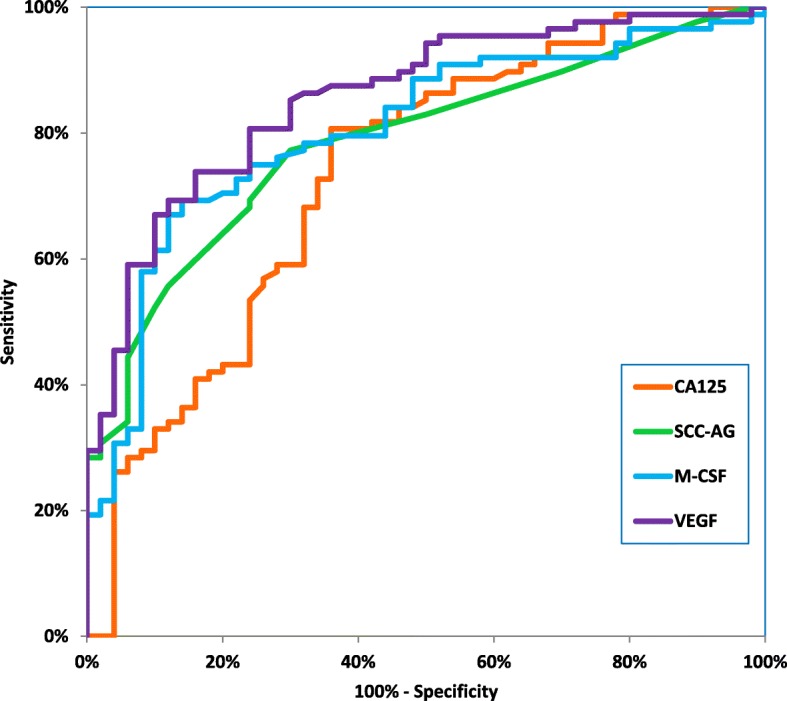
Diagnostic criteria of ROC curve for tested parameters in squamous cell cervical cancer group

**Fig. 5:**
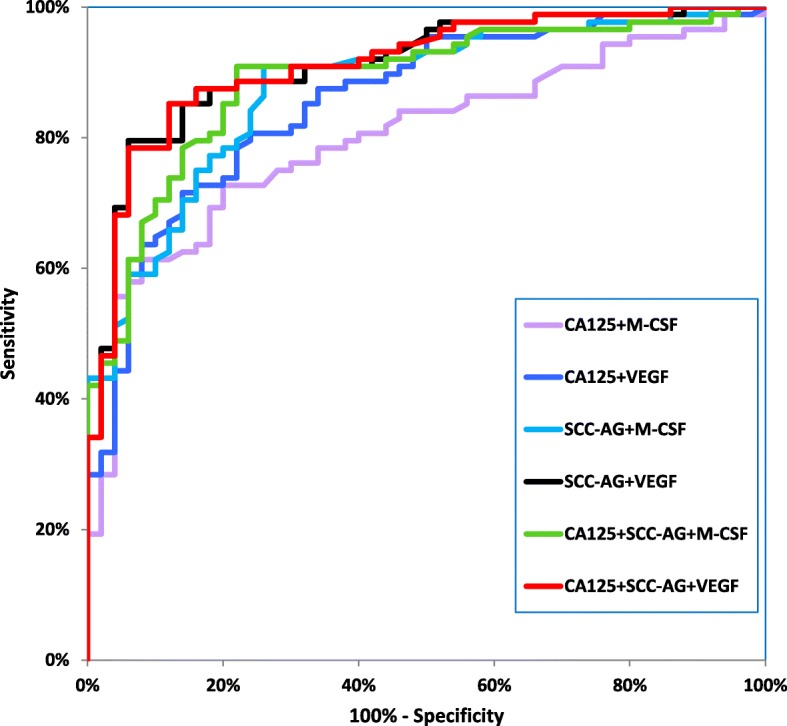
Diagnostic criteria of ROC curve for tested parameters in combination with CA 125 and SCC-Ag in squamous cell cervical cancer group

Table 4 shows the following diagnostic criteria: sensitivity (SE) and specificity (SP), in patients with the two histological types of cervical cancer-squamous cell carcinoma and adenocarcinoma (Table 4). We indicated that the SE of tested parameters in the squamous cell carcinoma group was the highest for VEGF and SCC-Ag (81.18%)- higher than that for CA 125 (80.00%) and M-CSF (69.41%) (Fig. 2). When considering the adenocarcinoma group the highest SE value was presented by VEGF (86.67%), and the lowest by SCC-Ag (53.33%), while both CA 125 and M-CSF demonstrated 66.67% (Fig. 3). It is worth noting that in the both cervical cancer groups evaluated as one, the highest SE was also presented by VEGF (82.00%). As displayed in the Table 4, the highest SP value was demonstrated by M-CSF for squamous cell carcinoma group and for adenocarcinoma group (86.00%). The combined use of the tested parameters with CA 125 antigen or SCC-Ag resulted in an increase in SE, but it did not improve the SP in either of the two cervical cancer groups (Fig.4, Fig. 5). The highest values of diagnostic criteria were obtained for the combination of VEGF with CA 125 and SCC-Ag for squamous cell carcinoma, adenocarcinoma and total cervical cancer group.

**Table 4:** Diagnostic criteria of tested parameters and in combined analysis with CA 125 and Scc-Ag in cervical cancer patients

Tested parameters	Diagnostic criteria (%)	Cervical Cancer		
		*Squamous cell carcinoma*	*Adenocarcinoma*	TOTAL
M- CSF	SE	69.41%	66.67%	69.00%
	SP	86.00%	86.00%	86.00%
VEGF	SE	81.18%	86.67%	82.00%
	SP	76.00%	76.00%	76.00%
CA 125	SE	80.00%	66.67%	78.00%
	SP	68.00%	68.00%	68.00%
SCC-Ag	SE	81.18%	53.33%	77.00%
	SP	74.00%	74.00%	74.00%
M-CSF + CA 125	SE	91.76%	86.67%	91.00%
	SP	66.00%	66.00%	66.00%
M-CSF + SCC-Ag	SE	91.76%	86.67%	91.00%
	SP	66.00%	66.00%	66.00%
M-CSF + CA 125 + SCC-Ag	SE	98.82%	93.33%	98.00%
	SP	44.00%	44.00%	44.00%
VEGF+CA 125	SE	95.29%	93.33%	95.00%
	SP	52.00%	52.00%	52.00%
VEGF+SCC-Ag	SE	96.47%	93.33%	96.00%
	SP	60.00%	60.00%	60.00%
VEGF+CA 125 + SCC-Ag	SE	100.00%	100.00%	100.00%
	SP	36.00%	36.00%	36.00%

The relationship between the diagnostic SE and SP is illustrated by the ROC curve. The AUC indicates the clinical applicability of a tumor marker as a diagnostic tool. Table 5 shows the results of our in-depth analysis of the AUC for all the studied biomarkers separately and in different combinations in the two examined cervical cancer groups (Table 5). The VEGF area under the ROC curve was the largest in the both adenocarcinoma group and squamous cell carcinoma (0.9082 and 0.8566). Interestingly, AUC values for CA 125 were the lowest (0.7340 and 0.6309, respectively) among all tested parameters. Considering the squamous cell carcinoma group, AUC of all the tested parameters was significantly larger in comparison to AUC = 0.5 (borderline of the diagnostic usefulness of the test) (*p* < 0.001 in all cases), instead of adenocarcinoma group where the AUC of M-CSF did not reach statistical significance comparing to AUC = 0.5 (*p* = 0.0762). Additionally, the combination of M-CSF or VEGF with CA 125 demonstrated AUC similar to that of these parameters separately. The addition of SCC-Ag to diagnostic panel improved the AUC value in both groups. VEGF in conjunction with both conventional tumor markers achieved the closest results to histopathological diagnosis as a marker of squamous cell carcinoma (0.9120) and adenocarcinoma (0.9509).

**Table 5:** Diagnostic criteria of ROC curve for tested parameters and CA 125 and SCC-Ag

Tested parameters	*Squamous cell carcinoma*				Adenocarcinoma			
	AUC	SE	95% C.I. (AUC)	*p* (AUC = 0.5)	AUC	SE	95% C.I. (AUC)	*p* (AUC = 0.5)
M-CSF	0.8051	0.0383	(0.730–0.880)	**<0.001**	0.6973	0.1113	(0.479–0.915)	0.0762
VEGF	0.8566	0.0321	(0.794–0.920)	**<0.001**	0.9082	0.0447	(0.821–0.996)	**<0.001**
CA 125	0.7340	0.0461	(0.644–0.824)	**<0.001**	0.6309	0.0974	(0.440–0.822)	**0.0179**
SCC-Ag	0.7866	0.0383	(0.711–0.862)	**<0.001**	0.8018	0.0765	(0.652–0.952)	**<0.001**
M-CSF+ CA 125	0.8006	0.0376	(0.727–0.874)	**<0.001**	0.7164	0.1121	(0.497–0.936)	0.0536
M-CSF+ SCC-Ag	0.8760	0.0296	(0.818–0.934)	**<0.001**	0.8427	0.0698	(0.706–0.980)	**<0.001**
M-CSF+ CA 125+ SCC-Ag	0.8869	0.0287	(0.831–0.943)	**<0.001**	0.8464	0.0692	(0.711–0.982)	**<0.001**
VEGF+ CA 125	0.8576	0.0322	(0.795–0.921)	**<0.001**	0.9127	0.0417	(0.831–0.995)	**<0.001**
VEGF+ SCC-Ag	0.9109	0.0250	(0.862–0.960)	**<0.001**	0.9445	0.0320	(0.882–1.005)	**<0.001**
VEGF+ CA 125+ SCC-Ag	0.9120	0.0249	(0.863–0.961)-	**<0.001**	0.9509	0.0286	(0.895–1.007)	**<0.001**

## Discussion

Great efforts have been made to identify novel biomarkers aiming at improving the detection of the invasive cervical cancer at the earliest possible stage [2]. Such tumor markers will be molecules arising from the presence of a tumor, which can appear in the surrounding tissue, and then within the blood [11, 13]. Thus, we hypothesized that the cytokines participating in angiogenesis and tumor invasion may be useful in early detection of the cancerous changes. The studied parameters appear to be effective in potential diagnosis of the analyzed histotypes of cervical cancer [39–41]. Although we did not observed any significant changes in the analyzed serum markers between the squamous cell carcinoma and adenocarcinoma group, which is consistent with other study [42], all the analyses were performed separately for the two groups. In this research, we demonstrated significantly higher plasma concentrations of VEGF, M-CSF, CA 125 and SCC-Ag in the squamous cell carcinoma group and VEGF, M-CSF, SCC-Ag in the adenocarcinoma group compared to the healthy women.

Comparable results for VEGF were obtained by Srivastava et al. [43], Zusterzeel et al. [42] and Du et al. [44] where positive correlation between the serum VEGF level, tumor stage and its size was found. On the other hand, Katanyoo et al. demonstrated that the pretreatment serum levels of VEGF in cervical cancer patients do not correlate with stage and tumor characteristic [45]. This discrepancy between the results is probably the effect of different composition and size of the studied groups. Applicability of serum VEGF has been confirmed in diagnosis of gastric [46], liver [47], colorectal [48], lung [49], prostate [50, 51], breast [20, 21, 52], ovarian cancer [27, 53, 54]. The literature data suggest that VEGF can be serum tumor marker in general regardless of its location, but additional analyzes are needed due to the ambiguity of results. Moreover, looking at the available results, attention should be paid to the insufficient sensitivity of this parameter and thus, the necessity to use a panel with the specific commonly known tumor marker. In present study the combination of VEGF with CA 125 or SCC-Ag significantly improved the diagnostic sensitivity.

Hematopoietic cytokines participate in hematopoiesis regulation, but they also appear to play a crucial role in the development of cancers e.g. increased levels of M-CSF in ovarian [53, 55], endometrial [56], breast [52, 57–59] and cervical cancer or cervical intraepithelial neoplasia patients [60]. In this study, M-CSF plasma concentrations were significantly higher compared to control in case of both squamous cervical cancer adenocarcinoma group. In case of diagnostic criteria of cancer, M-CSF demonstrated comparable SE and SP in two studied histological types of cervical cancer.

Porika et al. found that serum SCC-Ag levels in squamous cell carcinoma and CA 125 levels in adenocarcinoma patients were correlated with clinical stage and lymph node metastasis, but no association was observed between the marker levels, tumor size and patient age, concluding that SCC-Ag and CA 125 are relatively specific for the squamous cell carcinoma of cervix and the adenocarcinoma of cervix, respectively [61]. However, in our study, we demonstrated no variations in CA 125 concentrations between adenocarcinoma patients, dysplasia patients and controls, which can be explained by small study group size. It is worth noting that there is limited data evaluating the clinical applicability of serum preoperative CA 125 concentration measurement in patients with cervical adenocarcinoma. Duk et al. found that CA 125 plasma level elevations are correlated with advanced FIGO stage, disease progression, and survival [62] which was confirmed by Bender et al. [63] and Kotowicz et al. [64].

In this work plasma concentrations of SCC-Ag were significantly higher in the cervical cancer group compared to the healthy controls. Our data is in agreement with the results of other researchers regarding the diagnostic usefulness of antigen SCC in this malignancy [65, 66]. Moreover, its prognostic significance, both for the recurrence-free and overall survival, has been confirmed by other researchers in the early stages of cervical cancer [67, 68]. Considering squamous cell cervical cancer group, the highest SE and SP was obtained for both SCC-Ag and CA 125 simultaneously and M-CSF, respectively. Suzuki et al. demonstrated that preoperative serum measurement of M-CSF combined with SCC-Ag can be selective diagnostic marker for squamous cell carcinoma arising in mature cystic teratoma of the ovary [69]. However, in our study, the greatest AUC for this type of cancer was obtained for VEGF, which is in agreement with study performed by Lebrecht et al. [70]. Analysis showed that VEGF in conjunction with SCC-Ag and CA 125 demonstrated the highest SE when assessed together. Nevertheless, some studies state that the most useful serum marker for squamous cell cervical cancer diagnosis is still SCC-Ag [71, 72]. However, our research clearly indicated that M-CSF demonstrated higher diagnostic power in squamous cell carcinoma group compared to the commonly used tumor markers- SCC-Ag and CA 125.

The accuracy of Pap smear in diagnosing cervical precancerous lesions remain low. Sensitivity and specificity of Pap smear in diagnosing cervical dysplasia vary from 34.3 to 93.8% and from 34.7 to 96.5%, respectively [73–77]. Cytology is subjective with poorly reproducible criteria and a trade-off between sensitivity and specificity should be emphasized. A high misdiagnosis percentage of uterine cervical cancer patients results from the high rate of false negative cervical cytology results [78, 79]. Due to this risk of error, there is clearly a need to search for new techniques and markers, which sensitivity will be higher in comparison to the methods currently used. The purpose of the new markers is primarily to reduce the percentage of undiagnosed patients, which will also improve their survival rate. Cytological screening proves successful for squamous lesions [2, 80] however this method is not effective in diagnostics of cervical adenocarcinoma yet [40]. Moreover, the cervical adenocarcinoma seems to be aggressive, and more often, lymph nodes metastases are observed in these patients [1]. A number of studies concerning cervical adenocarcinoma have addressed the use of tumor markers for pretreatment evaluation of this disease. Among all the assessed parameters, VEGF was the only marker that demonstrated sufficient diagnostic sensitivity in cervical adenocarcinoma patients. Serum measurement of VEGF in conjunction with CA 125 and SCC-Ag demonstrated a high diagnostic power based on AUC. VEGF plays a crucial role in neoangiogenesis, thus influencing disease progression and metastasis, including cervical cancer patients [14, 16, 81].

The ROC curve, which is the SE/SP diagram still remains important criterion for tumor markers [38]. The larger AUC corresponds to a better tumor marker. In this study, the ROC area of VEGF was the largest from all the tested parameters in both histological groups. Additionally, we observed statistically significantly larger AUCs for the studied markers compared to AUC = 0.5 in squamous cell cervical cancer and for VEGF, CA 125 and SCC-Ag, but not for M-CSF in adenocarcinoma group. Combined analysis showed that panel consisting of VEGF, CA 125, and SCC-Ag demonstrated the highest diagnostic power in both squamous cell carcinoma and adenocarcinoma groups.

## Conclusions

Early detection of cervical cancer in patients is of utter importance. All the studied parameters fail in basic cervical cancer screening when considered separately. In our research the greatest diagnostic value is demonstrated when combined diagnostic panel of tumor markers is used. However, what should be emphasized is that the exploratory analysis need to be confirmed in separate future follow-up studies.

## Abbreviations

CA 125: Cancer Antigen 125
CIN: Cervical Intraepithelial Neoplasia
CMIA: Chemiluminescent Microparticle Immunoassay
CT: Computed Tomography
CV%: Coefficient of Variation
ELISA: Enzyme-Linked Immunosorbent Assay
FIGO: International Federation of Gynecology and Obstetrics
HPV: Human Papilloma Virus
M-CSF: Macrophage-Colony Stimulating Factor
M-CSFR: Macrophage-Colony Stimulating Factor Receptor
ROC: Receiver Operator Characteristic
SCC-Ag: Squamous Cell Carcinoma Antigen
TAMs: Tumor Associated Macrophages
VEGF: Vascular Endothelial Growth Factor
